# Instrumental Rotation for Persistent Fetal Occiput Posterior Position: A Way to Decrease Maternal and Neonatal Injury?

**DOI:** 10.1371/journal.pone.0078124

**Published:** 2013-10-18

**Authors:** Fabien Vidal, Caroline Simon, Christelle Cristini, Catherine Arnaud, Olivier Parant

**Affiliations:** 1 CHU Toulouse, Pole de Gynécologie Obstétrique, Hôpital Paule de Viguier, Toulouse, France; 2 CHU Purpan Unité de soutien à la recherche, Toulouse, France; 3 INSERM, U.1027, France; 4 Université Paul-Sabatier, Toulouse, France; University of Tennessee Health Science Center, United States of America

## Abstract

**Objective:**

To evaluate immediate perineal and neonatal morbidity associated with instrumental rotations performed with Thierry’s spatulas for the management of persistent posterior occiput (OP) positions.

**Methods:**

Retrospective study including all persistent occiput posterior positions with vaginal OP delivery, from August 2006 to September 2007. Occiput anterior deliveries following successful instrumental rotation were included as well. We compared maternal and neonatal immediate outcomes between spontaneous deliveries, rotational and non rotational assisted deliveries, using χ^2^ and Anova tests.

**Results:**

157 patients were enrolled, comprising 46 OP spontaneous deliveries, 58 assisted OP deliveries and 53 deliveries after rotational procedure. Instrumental rotation failed in 9 cases. Mean age and parity were significantly higher in the spontaneous delivery group, while labor duration was shorter. There were no significant differences in the rate of severe perineal tears and neonatal adverse outcomes between the 3 groups.

**Conclusion:**

Instrumental rotation using Thierry’s spatulas was not associated with a reduced risk of maternal and neonatal morbidity for persistent OP deliveries. Further studies are required to define the true interest of such procedure in modern obstetrics.

## Introduction

Persistent occiput posterior (OP) position is the most common malposition at delivery, with an incidence ranging from 2 to 13% [1,2]. To date, it is still unclear if OP presentations originate prenatally (or in early labor) [3] or result from a malrotation from occiput anterior (OA) or occiput transverse (OT) positions [4]. They are associated with prolonged labor, increased rates of operative vaginal and cesarean deliveries and higher risk of maternal and neonatal adverse outcomes [1,2,5]. Therefore, fetal head rotation from OP to OA has been proposed to decrease morbidity.

Sims' posture consists in a maternal lateral recumbent during labor on the same side as the fetal spine. It might enhance rotation to OA position and hence reduce incidence of cesarean deliveries [6]. Nevertheless, it is not recommended by the French College of Obstetricians due to a lack of evidence [7]. 

Manual rotation is a common and effective technique to decrease cesarean delivery rate in patients presenting with persistent OP position during labor [8]. The success of the procedure depends on maternal age and parity, cervical dilatation and indication for rotation. However its failure rate is high, ranging from 10 to 26%. 

Several studies have described an increase of adverse outcomes for both mother and baby associated with instrumental rotations (IR) [9-11]. Therefore the use of rotational forceps is still debated and has been prohibited in many maternity wards. Conversely, others have suggested IR to be a safe option for the management of persistent OP positions, yielding decreased maternal morbidity rates [12]. 

**Figure 1:**
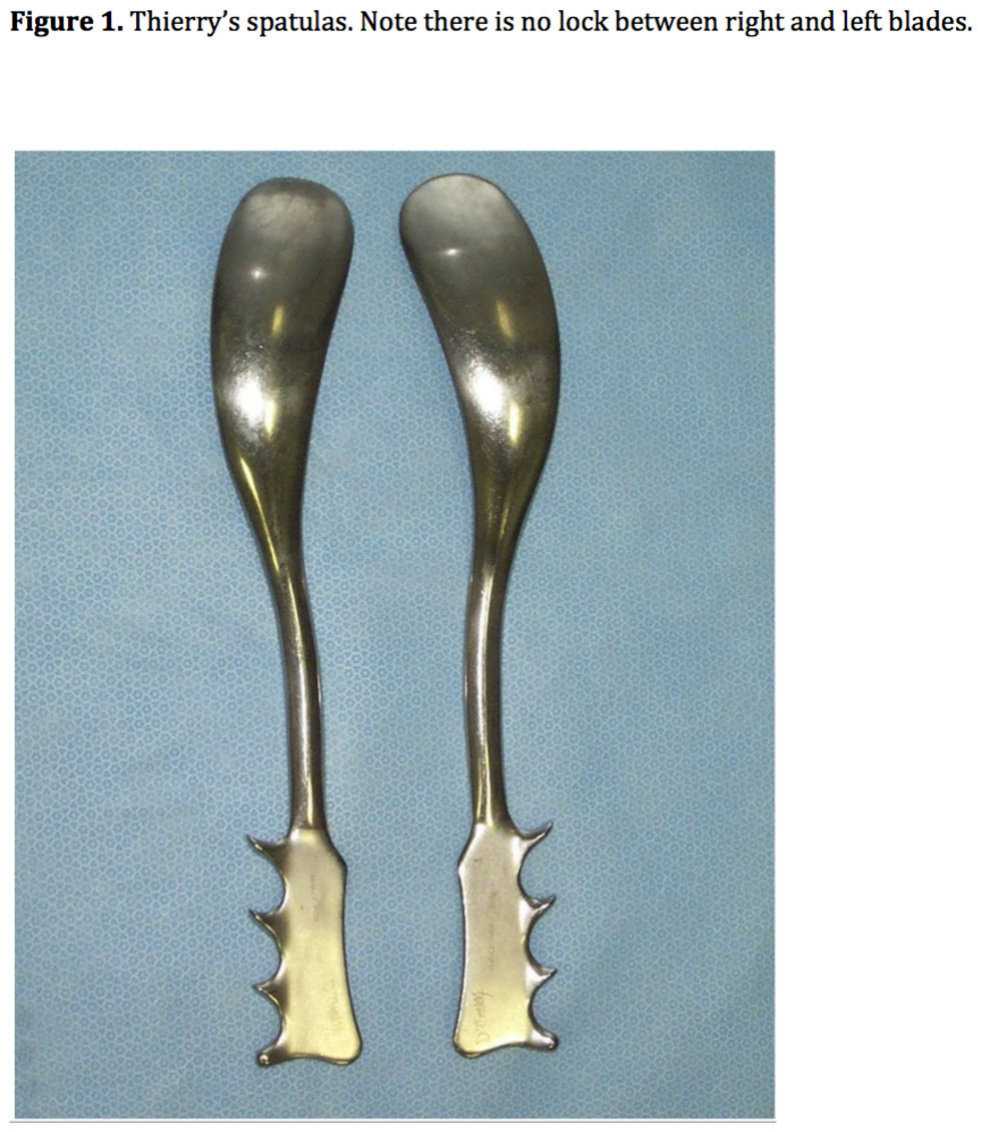
Thierry' spatulas. Note there is no lock between right and left blades.

Designed in 1950, Thierry’s spatulas (TS) are made of 2 independent control spoons (Figure 1). The blades have a large cephalic curve that prevents excessive traction on the fetal head. Their minimal pelvic curve may be compatible with fetal head rotation. Their mechanism of action is based on direction and propulsion rather than traction. They propel the fetus through the birth canal in the suboccipitomental axis, taking support laterally on maternal perineum and medially on fetal malar bones. TS are commonly used in our maternity ward for operative deliveries and IR, with good efficiency. However, specific data assessing the use of TS in IR are lacking.

Therefore, we aimed to evaluate the immediate perineal and neonatal morbidity associated with IR performed with TS in comparison to non-rotational forceps and spontaneous deliveries in women diagnosed with persistent OP position.

## Patients and Methods

### Study design

From August 2006 to September 2007, every persistent OP positions followed by OP vaginal delivery were enrolled in this retrospective study, including spontaneous (SD) and assisted deliveries (AD). OA deliveries following IR procedure were included as well. Study population was stratified in 3 subgroups according to the type of delivery: spontaneous deliveries (SD group), assisted deliveries without IR (AD group) and deliveries after IR (IR group). Exclusion criteria were non-singleton births, OA and cesarean deliveries. OA deliveries following manual rotation for persistent OP position were also excluded. 

The study received agreement of the regional institutional review board.

### Obstetrical protocol

All SD were performed by a midwife. Instrumental extractions were managed by an attending physician or by a senior resident, on persistent OP position from +2 to +4 stations. Extraction modalities (rotational or non rotational) were at the attending physician's discretion. TS were the only instrument used to assist delivery during the study period.

### Instrumental rotation

Diagnosis of fetal head position had to be certain before IR was performed. At the time of inclusion, only clinical examination was recommended in our department. Ultrasound scan examination was thus performed only when required. Deliveries following IR were either spontaneous or assisted. In this latter case, TS were removed after rotational procedure and replaced in order to assist fetal extraction. The decision concerning the type of delivery depended on the obstetrician in charge. IR was considered successful when fetal head was in OA position at delivery. 

### Maternal and neonatal parameters

Our primary outcome was to compare immediate maternal and neonatal morbidity according to the type of delivery. 

Maternal morbidity parameters included episiotomy rate, incidence of perineal and genital lacerations, perineal hematoma and postpartum hemorrhage. Postpartum hemorrhage was defined as a blood loss following delivery greater than 500mL. In women who underwent an episiotomy, genital lacerations corresponded to additional tears or the worsening of injuries related to episiotomy.

Neonatal morbidity parameters included Apgar score, umbilical arterial and venous pH values, major and minor fetal injuries and neonatal intensive care unit admissions. Umbilical cord gases acidemia corresponded to artery pH values less than 7.1. Birth trauma was defined as a composite of skull fracture, cerebral hematoma, facial nerve palsy and clavicular fracture.

### Statistical analysis

All analyses were performed using Stata Statistical Software (release 9.0; Stata Corporation, College Station, TX). Mean and standard deviation (Sdev) were used for quantitative variables normally distributed. Otherwise we used median and interquartile range (IQR). To compare the three groups defined by mode of delivery (SD, AD and IR groups), the Chi-square or Fisher exact tests were used for categorical variables and ANOVA or Kruskall-Wallis tests were used for quantitative variables. A *p* value of less than 0.05 was considered significant.

## Results

### Population stratification

**Figure 2:**
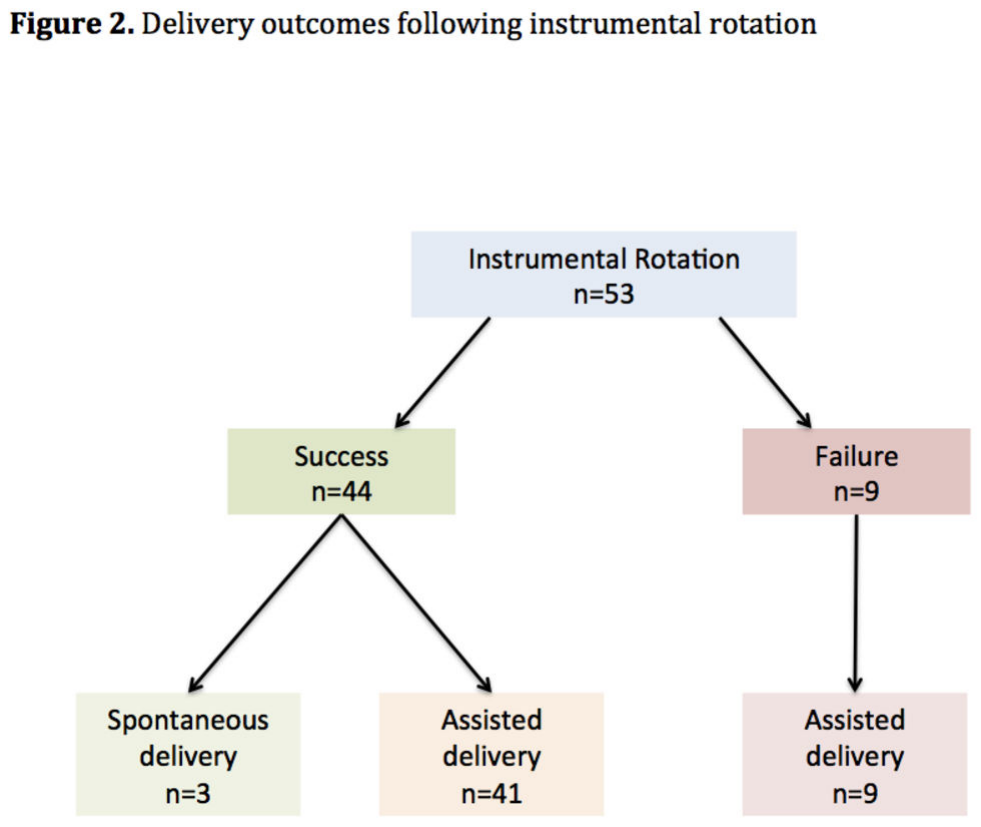
Delivery outcomes following instrumental rotation.

Among the 4490 deliveries occurring during our study period, 157 patients matched our inclusion criteria (3.5%). Among the study population, 104 (66.2%) patients delivered in OP position without attempt of rotational procedure: 46 (29.3%) deliveries were spontaneous (SD group) and 58 (36.9%) were assisted (AD group). Noteworthy, the diagnosis of OP presentation was made at the time of delivery for 28 (SD) and 44 (AD) patients. Fifty-three (33.8%) patients delivered after IR. Indications for instrumental delivery were mainly failure of progress (59.4%), fetal bradycardia (19.8%) or both (18.9%).

### IR accuracy (Figure 2)

IR resulted in OA delivery in 44/53 cases (83%). Failure in the procedure systematically led to operative delivery in OP position. Conversely, successful rotations were subsequently followed by instrumental (n=41) or spontaneous deliveries (n=3). In the IR group, diagnosis of persistent OP presentation required ultrasound examination in 14 patients (26.4%) to confirm clinical hypothesis. 

### Maternal and labor characteristics (Table 1)

**Table 1:** Maternal and obstetrical demographics.

**Characteristics**	**SD group** n=46	**AD group** n=58	**IR group** n=53	***p*-value**
**Age**, mean (Sdev)	31.3 (4.6) ^A^	29 (4.6) ^B^	29.2 (5.1) ^A^	0.039
**BMI**				
Med (IQR)	21.8 (20.1-23.7)	22.5 (20.2-24.9)	21.4 (19.8-24.1)	0.413
> 30, n (%)	2 (4.3)	3 (5.2)	1 (1.9)	0.533
**Median Parity** (IQR)	1 (0-1) ^A^	0 (0-1) ^B^	0 (0-0) ^B^	<10^-4^
**Prior cesarean**, n (%)	0 (0.0)	6 (10.3)	3 (5.7)	0.078
**Gestational age**, med (IQR)	39.5 (39-40.2)	40 (39-41)	40 (38.5-41)	0.498
**Labor duration** (min)				
First stage, med (IQR)	175 (100-250) ^A^	245 (150-400) ^B^	300 (180-420) ^B^	0.002
Active phase, med (IQR)	14.5 (10-25) ^A^	25 (15-34) ^B^	23 (15-30) ^B^	0.002
**PP hemorrhage**, n (%)	0 (0.0)	4 (6.9)	2 (3.77)	0.244

A ^B^ there is a statistical significance in the comparison between the groups marked with a different letter

Med (IQR) : median (interquartile range)PP : postpartum

 Epidural anesthesia was largely employed (94.9%). There were no significant differences between the 3 groups regarding BMI, prior c-section rate and gestational age. Patients with SD presented with a higher mean parity compared to AD and IR groups (*p*<10^-4^). First stage and active phase of labor were statistically longer in AD and IR patients. Postpartum hemorrhage occurred in 6 patients of the AD (n=4) and IR (n=2) groups, however, comparison with SD group reached no statistical significance. Most of the bleedings (83.3%) were controlled by intra-venous administration of oxytocin and sulprostone. One hemorrhage required invasive procedures, including surgery and embolization. 

### Perineal outcomes (Table 2)

**Table 2:** Perineal outcomes.

**Outcomes**	**SD group** n=46	**AD group** n=58	**IR group** n=53	***p*-value**
**Episiotomy**, n (%)	18 (39.1) ^A^	56 (96.5) ^B^	50 (94.3) ^B^	<10^-4^
**Perineal laceration**, n (%)				
None	24 (52.2) ^A^	49 (84.5) ^B^	50 (94.3) ^B^	<10^-4^
1^st^ degree	18 (39.1)	4 (6.9)	2 (3.8)	
2^nd^ degree	3 (6.5)	3 (5.2)	0 (0.0)	
3^rd^ degree	1 (2.2)	2 (3.4)	1 (1.9)	
4^th^ degree	0 (0.0)	0 (0.0)	0 (0.0)	
**Cervical laceration**, n (%)	0 (0.0)	0 (0.0)	2 (3.8)	0.197
**Vaginal laceration**, n (%)	6 (13.0)	5 (8.6)	2 (3.8)	0.251
**Perineal hematoma**, n (%)	0 (0.0)	0 (0.0)	2 (3.8)	0.5

A ^B^ there is a statistical significance in the comparison between the groups marked with a different letter

The episiotomy rate was 79% and significantly associated to instrumental deliveries (*p*<10^-4^). 34 patients suffered from perineal tears (21.7%), mostly after SD (*p*<10^-4^). Nevertheless, there was no significant difference in the incidence of severe perineal lacerations between the 3 groups. They occurred in 4 patients (2.5%) and were all third-degree lacerations. All cervical tears and perineal hematomas occurred in the IR groups. All perineal hematomas were managed by surgery and 1 required additional embolization of a perineal artery. 

### Neonatal outcomes (Table 3)

There was no difference in neonates median weights between the 3 groups. Shoulder dystocia occurred in 1 case, after AD, but yielded no adverse consequences. There was no significant difference in the incidence of umbilical cord gases acidemia and low Apgar score at delivery. However, the only case of 5-minute Apgar score less than 7 was observed after SD. 

As expected, IR were associated with a significant increase of cutaneous injury rate compared to SD (*p*=0.018). In particular, we report 3 cases of sub cutaneous hematomas and 1 case of skin wound that did not cause any esthetic damage. However, no significant difference was observed in comparison to AD group. No birth trauma occurred.

**Table 3:** Neonatal outcomes, according to the type of delivery : spontaneous (SD), assisted without instrumental rotation (AD), and after instumental rotation (IR) with and without assisted delivery.

**Outcomes**	**SD group** n=46	**AD group** n=58	**IR group** n=53	***p*-value**
**Weight**, grams, med (IQR)	3255 (3080-3530)	3260 (3030-3610)	3260 (2960-3520)	0.977
**Apgar < or = 7**, n (%)				
1 minute	3 (6.5)	5 (8.6)	7 (13.2)	0.551
5 minutes	1 (2.2)	53 (93.0)	49 (94.2)	0.293
**PHAo**, n (%)				
<7.1	2 (4.6)	4 (7.0)	3 (5.8)	0.915
> or = 7.1	41 (95.3)	53 (93.0)	49 (94.2)	
**Cutaneous injuries**, n (%)				
None	46 (100) ^A^	48 (82.8) ^B^	44 (83) ^B^	0.018
Bruise	0 (0.0)	8 (13.8)	5 (9.4)	
Hematoma	0 (0.0)	2 (3.4)	3 (5.7)	
Wound	0 (0.0)	0 (0.0)	1 (1.9)	
**Birth trauma**, n	0	0	0	-
**Pediatric transfer**, n (%)	0 (0.0)	5 (8.6)	4 (7.6)	0.098
neonatalogy unit	0 (0.0)	4 (6.9)	4 (7.6)	
intensive care unit	0 (0.0)	1 (1.7)	0 (0.0)	

A ^B^ there is a statistical significance in the comparison between the groups marked with a different letter

Med (IQR) : median (interquartile range)

Pediatric unit transfers were only observed in AD and IR groups and concerned 9 neonates. One suffered from a diaphragmatic hernia and required intensive care nursery. Four presented a moderate prematurity (from 33 to 34.5 weeks’ gestation) and were transferred to pediatric unit for non-invasive ventilation and enteral nutrition. No short-term adverse outcomes occurred and hospital stay did not exceed 10 days. There were no preterm births in the SD group. Four neonates were transferred due to slight respiratory distress independently of any prematurity. All neonates recovered a normal lung function within 2 days following admission and none required invasive ventilation.

## Discussion

Our study supports that instrumental rotations (IR) performed for persistent OP positions with Thierry’s Spatulas (TS) are efficient and not associated with poorer outcomes compared to spontaneous deliveries (SD) and assisted deliveries without rotational procedure (AD) regarding immediate maternal and neonatal morbidity. 

Few studies focusing on TS are available, mainly because they are not spread in many institutions worldwide [13-15]. However, TS are commonly used by French obstetricians and continue to be taught in several maternity wards. Their efficiency regarding OP position management is still debated [16,17]. In a prospective cohort of primiparous women, TS allowed fetal extraction in all cases, independently of fetal head position [14]. 

Mediolateral episiotomy was largely performed in patients with instrumental deliveries (95.5%) while its rate was 39.1% in SD group. Recent prospective studies failed to demonstrate that routine episiotomy was responsible for increased anal sphincter tearing in operative vaginal deliveries [18,19]. Restrictive use was associated with less post partum hemorrhage and perineal infections. Episiotomy should not be performed routinely, as supported by the 2006 French College of Obstetricians guidelines [20]. However, in a 2008 British survey, two-thirds of obstetricians held the view that routine use of episiotomy decreased the likelihood of severe perineal lacerations in forceps delivery, suggesting that it would take time for general practice to evolve [21]. Our study period started the first year following publication of the French guidelines, thus the high rate of episiotomies. Noteworthy, routine episiotomy was only associated with instrumental extractions. Similarly, high rates of episiotomy have been reported in all studies focusing on TS. Beyond old habits, the mechanism of action of TS may partially explain the rate of episiotomy. Contrary to conventional forceps, TS comprise 2 independent spoons that are moved aside to propel fetal head, resulting in tensing perineum. Obstetricians might thus be more liable to perform an episiotomy. 

Within the study population, primiparous were predominant and persistent OP position was associated with a high rate of operative deliveries (70.7%), similar to other studies [1,5]. As expected, median parity was significantly higher in patients that delivered spontaneously. Primiparity, assisted vaginal deliveries and OP positions have been shown to be independent risk factors for severe perineal lacerations [5]. Nevertheless, we observed a very low incidence of severe perineal tears (2.5%) and no fourth-degree laceration occurred. Surprisingly, previous studies have reported higher rates of severe perineal tears associated with TS extractions for persistent OP positions, ranging from 8.2% to 17.4% [13,22]. We have no formal explanation for such discrepancy. Operative delivery reports were conscientiously filled in and data collection was achieved right after delivery. True extent of perineal injury might have been under-staged. However, we always carefully control cervical, vaginal and perineal areas after instrumental extractions. 

Rotational forceps > 45° for persistent OP positions may increase traumatic neonatal outcomes [23,24]. Hankins et al. have reported a 9.7% rate of severe injuries, including facial and brachial nerves tears and subdural hematoma [24]. Conversely, vacuum extractors improve fetal head rotation and may reduce OP delivery rate. They are associated with less immediate maternal complications, while long-term morbidity (pelvic floor dysfunction) is not reduced [25]. However, vacuum extractors are also associated with severe potential complications [26] and have been banished by the Society of Obstetricians and Gynecologists of Canada [27].

In few reports, paradoxically, rotational forceps are described as a safe procedure when performed by experienced physicians. In a retrospective cohort of 267 patients, Feldman et al. provide comparative outcomes between rotational and non-rotational forceps deliveries [12]. Indeed, IR performed with Leff’s forceps led to decreased rates of episiotomy and severe perineal lacerations and was associated with shorter duration of second stage of labor. Furthermore, Al-Suhel et al. have shown that prudent use of Kjelland's forceps for fetal head rotation was responsible for a very low rate of perinatal adverse outcomes, indistinguishable from non rotational vacuum extractor [28]. Obstetrician experience strongly participates in reducing morbidity associated with IR, and the type of instrument probably represents an additional important parameter. Consequently, beyond the procedure itself, we have to determine which forceps are convenient for IR.

Regarding neonatal morbidity, the comparison should be restricted to IR and AD, since SD were likely the easier deliveries. Indeed, cutaneous injuries only occurred after assisted extractions. Similarly, all the transfers to pediatric unit were observed after instrumental deliveries, with no significant difference between AD and IR. It should be emphasized that every preterm birth among the study population required assistance for delivery. Beyond the bias it conveys, this finding highlights the fact that the association of prematurity and failure of progress or abnormalities in the fetal heart monitoring prompted the obstetrician to accelerate the delivery. 

TS have been evaluated once in IR. In a preliminary study on primiparous, Parant et al. included 49 persistent OP vaginal deliveries and found no significant difference between AD and IR group regarding several perineal tears and neonatal morbidity [22]. In our study, incidence of third degree lacerations was similar in the 3 groups and perineal hematoma only occurred after IR, with no statistical significance. We have found no difference in neonatal morbidity. Therefore, we cannot conclude that patients and neonates would benefit from IR. 

In conclusion, the debate surrounding IR for persistent OP presentations continues, as we seek to optimize outcomes for both mother and baby. The literature supports the hypothesis that the safety profile of an instrument is largely determined by the experience of the clinician with that particular instrument, although it is likely that some instruments are inherently more prone to causing birth injury than others. In this retrospective study of Thierry’s spatulas rotations for persistent OP presentations, we found an increase of morbidity in cutaneous injuries to the neonate, and an increase in pediatric unit transfers when compared to spontaneous deliveries. However, when compared to assisted deliveries, instrumental rotations were not associated with increased morbidity. With the data presented here, it is now possible to design larger prospective studies to determine if use of Thierry’s spatulas will benefit or harm mother and new born infant. 

## References

[1] Cheng YW, Shaffer BL, Caughey AB (2006) The association between persistent occiput posterior position and neonatal outcomes. Obstet Gynecol 107: 837-844. doi:10.1097/01.AOG.0000206217.07883.a2. PubMed: 16582120. 16582120

[2] Yancey MK, Zhang J, Schweitzer DL, Schwarz J, Klebanoff MA (2001) Epidural analgesia and fetal head malposition at vaginal delivery. Obstet Gynecol 97: 608-612. doi:10.1016/S0029-7844(00)01230-8. PubMed: 11275036. 11275036

[3] Akmal S, Tsoi E, Howard R, Osei E, Nicolaides KH (2004) Investigation of occiput posterior delivery by intrapartum sonography. Ultrasound Obstet Gynecol 24: 425-428. doi:10.1002/uog.1064. PubMed: 15343598. 15343598

[4] Gardberg M, Laakkonen E, Sälevaara M (1998) Intrapartum sonography and persistent occiput posterior position: a study of 408 deliveries. Obstet Gynecol 91: 746-749. doi:10.1016/S0029-7844(98)00074-X. PubMed: 9572223. 9572223

[5] Benavides L, Wu JM, Hundley AF, Ivester TS, Visco AG (2005) The impact of occiput posterior fetal head position on the risk of anal sphincter injury in forceps-assisted vaginal deliveries. Am J Obstet Gynecol 192: 1702-1706. doi:10.1016/j.ajog.2004.11.047. PubMed: 15902181. 15902181

[6] Wu X, Fan L, Wang Q (2001) [Correction of occipito-posterior by maternal postures during the process of labor]. Zhonghua Fu Chan Ke Za Zhi 36: 468-469. PubMed: 11758180. 11758180

[7] (2008) [Instrumental extractions. Guidelines]. J Gynecol Obstet Biol Reprod (Paris) 37 Suppl 8: S297-S300. doi:10.1016/S0368-2315(08)74767-7. 19731414

[8] Shaffer BL, Cheng YW, Vargas JE, Caughey AB (2010) Manual rotation to reduce caesarean delivery in persistent occiput posterior or transverse position. J Matern Fetal Neonatal Med. 10.3109/14767051003710276 20350240

[9] Jain V, Guleria K, Gopalan S, Narang A (1993) Mode of delivery in deep transverse arrest. Int J Gynecol Obstet 43: 129-135. doi:10.1016/0020-7292(93)90319-R. PubMed: 7905427. 7905427

[10] Menticoglou SM, Perlman M, Manning FA (1995) High cervical spinal cord injury in neonates delivered with forceps: report of 15 cases. Obstet Gynecol 86: 589-594. doi:10.1016/S0029-7844(95)80022-0. PubMed: 7675385. 7675385

[11] Ramin SM, Little BB, Gilstrap LC 3rd (1993) Survey of forceps delivery in North America in 1990. Obstet Gynecol 81: 307-311. PubMed: 8423970. 8423970

[12] Feldman DM, Borgida AF, Sauer F, Rodis JF (1999) Rotational versus nonrotational forceps: maternal and neonatal outcomes. Am J Obstet Gynecol 181: 1185-1187. doi:10.1016/S0002-9378(99)70105-5. PubMed: 10561642. 10561642

[13] Courtois L, Becher P, Maticot-Baptista D, Cour A, Zurlinden B et al. (2008) [Instrumental extractions using Thierry's spatulas: evaluation of the risk of perineal laceration according to occiput position in operative deliveries]. J Gynecol Obstet Biol Reprod (Paris) 37: 276-282. doi:10.1016/j.jgyn.2007.10.006. 18093747

[14] Parant O, Simon-Toulza C, Capdet J, Fuzier V, Arnaud C et al. (2009) [Immediate fetal-maternal morbidity of first instrumental vaginal delivery using Thierry's spatulas. A prospective continuous study of 195 fetal extractions]. Gynecol Obstet Fertil 37: 780-786. doi:10.1016/j.gyobfe.2009.07.010. PubMed: 19766049. 19766049

[15] Vanlieferinghen S, Girard G, Mandelbrot L (2009) [A comparison of maternal and fetal complications during operative vaginal delivery using Thierry's spatulas and the vacuum extractor]. J Gynecol Obstet Biol Reprod (Paris) 38: 648-654. doi:10.1016/j.jgyn.2009.09.015. 19896285

[16] Maisonnette-Escot Y, Riethmuller D, Chevriere S, Becher P, Floret N, et al. (2005) [Thierry's spatula instrumental extraction: a study of foetal-maternal morbidity]. Gynecol Obstet Fertil 33: 208-212 10.1016/j.gyobfe.2005.03.007 15894204

[17] Guyomar J (1965) [Obstetrical value of Thierry's spatules. Results of 1,000 applications]. Rev Fr Gynecol Obstet 60: 775-783. PubMed: 5852936. 5852936

[18] Murphy DJ, Macleod M, Bahl R, Goyder K, Howarth L et al. (2008) A randomised controlled trial of routine versus restrictive use of episiotomy at operative vaginal delivery: a multicentre pilot study. BJOG 115: 1693-1702; discussion: 10.1111/j.1471-0528.2008.01960.x 19035944

[19] Macleod M, Strachan B, Bahl R, Howarth L, Goyder K et al. (2008) A prospective cohort study of maternal and neonatal morbidity in relation to use of episiotomy at operative vaginal delivery. BJOG 115: 1688-1694. doi:10.1111/j.1471-0528.2008.01961.x. PubMed: 19035943. 19035943

[20] CNGOF (2006) [Episiotomy: recommendations of the CNGOF for clinical practice (December 2005)]. Gynecol Obstet Fertil 34: 275-279. doi:10.1016/j.gyobfe.2006.01.033. PubMed: 16622908. 16622908

[21] Macleod M, Murphy DJ (2008) Operative vaginal delivery and the use of episiotomy--a survey of practice in the United Kingdom and Ireland. Eur J Obstet Gynecol Reprod Biol 136: 178-183. doi:10.1016/j.ejogrb.2007.03.004. PubMed: 17459568. 17459568

[22] Parant O, Simon C, Capdet J, Tanguy Le Gac Y, Reme JM (2007) [Can we still perform instrumental rotations using Thierry's spatula? Preliminary study among primiparous]. J Gynecol Obstet Biol Reprod (Paris) 36: 582-587. doi:10.1016/j.jgyn.2007.03.013. 17499455

[23] (1994) Operative vaginal delivery. ACOG Technical. Bulletin Number 196-- August 1994 (replaces No. 152, February 1991). Int J Gynaecol Obstet 47: 179-185

[24] Hankins GD, Leicht T, Van Hook J, Uçkan EM (1999) The role of forceps rotation in maternal and neonatal injury. Am J Obstet Gynecol 180: 231-234. doi:10.1016/S0002-9378(99)70180-8. PubMed: 9914609. 9914609

[25] Vayssière C, Beucher G, Dupuis O, Feraud O, Simon-Toulza C et al. (2011) Instrumental delivery: clinical practice guidelines from the French College of Gynaecologists and Obstetricians. Eur J Obstet Gynecol Reprod Biol 159: 43-48. doi:10.1016/j.ejogrb.2011.06.043. PubMed: 21802193. 21802193

[26] Baerthlein WC, Moodley S, Stinson SK (1986) Comparison of maternal and neonatal morbidity in midforceps delivery and midpelvis vacuum extraction. Obstet Gynecol 67: 594-597. PubMed: 3960431. 3960431

[27] (2005) SOGC clinical practice guidelines. Guidelines for vaginal birth after previous caesarean birth. Number 155 (Replaces guideline Number 147), February 2005. Int J Gynecol Obstet 89: 319-331. doi:10.1016/j.ijgo.2005.03.015. 16001462

[28] Al-Suhel R, Gill S, Robson S, Shadbolt B (2009) Kjelland's forceps in the new millennium. Maternal and neonatal outcomes of attempted rotational forceps delivery. Aust N Z J Obstet Gynaecol 49: 510-514. doi:10.1111/j.1479-828X.2009.01060.x. PubMed: 19780735. 19780735

